# Multinutrients for the Treatment of Psychiatric Symptoms in Clinical Samples: A Systematic Review and Meta-Analysis of Randomized Controlled Trials

**DOI:** 10.3390/nu12113394

**Published:** 2020-11-04

**Authors:** Jeanette M. Johnstone, Andrew Hughes, Joshua Z. Goldenberg, Amy R. Romijn, Julia J. Rucklidge

**Affiliations:** 1 Helfgott Research Institute, National University of Natural Medicine, Portland, OR 97201, USA; jgoldenberg@nunm.edu; 2 Child and Adolescent Psychiatry, Oregon Health & Science University, Portland, OR 97239, USA; 3 Adult Psychiatry, Oregon Health & Science University, Portland, OR 97239, USA; hughean@ohsu.edu; 4 Australian Research Centre in Complementary and Integrative Medicine, Faculty of Health, University of Technology Sydney, Sydney 2007, Australia; 5 Department of Psychology, Swansea University, Swansea SA2 8PP, UK; a.r.romijn@swansea.ac.uk; 6 School of Psychology, Speech and Hearing, University of Canterbury, 8140 Christchurch, New Zealand

**Keywords:** systematic review, meta-analysis, multinutrients, vitamins, minerals, psychiatric symptoms, mood, depression, ADHD, autism

## Abstract

This systematic review and meta-analysis focused on randomized controlled trials (RCT) of multinutrients consisting of at least four vitamins and/or minerals as interventions for participants with psychiatric symptoms. A systematic search identified 16 RCTs that fit the inclusion criteria (*n* = 1719 participants) in six psychiatric categories: depression, post-disaster stress, antisocial behavior, behavioral deficits in dementia, attention-deficit/hyperactivity disorder, and autism. Grading of Recommendations, Assessment, Development and Evaluations (GRADE) was used to rate the evidence base. Significant clinical benefit was assessed using minimal clinically important differences (MIDs). Due to heterogeneity in participants, multinutrient formulas, outcome measures, and absence of complete data, only the Attention-Deficit/Hyperactivity Disorder (ADHD) category was eligible for meta-analyses. In ADHD populations, statistically and clinically significant improvements were found in global functioning, Mean Difference (MD) −3.3, *p* = 0.001, MID −3.26; Standardized Mean Difference (SMD) −0.49 *p* = 0.001 MD −0.5), clinician ratings of global improvement (MD −0.58, *p* = 0.001, MID −0.5) and ADHD improvement (MD −0.54, *p* = 0.002, MID −0.5), and clinician (but not observer) measures of ADHD inattentive symptoms (MD −1.53, *p* = 0.05, MID −0.5). Narrative synthesis also revealed a pattern of benefit for global measures of improvement, for example: in autism, and in participants with behavioral deficits in dementia. Post-natural disaster anxiety and the number of violent incidents in prison populations also improved. Broad-spectrum formulas (vitamins + minerals) demonstrated more robust effects than formulas with fewer ingredients. This review highlights the need for robust methodology-RCTs that report full data, including means and standard deviations for all outcomes-in order to further elucidate the effects of multinutrients for psychiatric symptoms.

## 1. Introduction

Poor nutrition is increasingly identified as a modifiable risk factor for the development and persistence of psychiatric problems [1], with research identifying that the Western diet, high in salt, saturated fat, ultra-processed ingredients, and sugar is most consistently associated with poor mental health [2,3]. Interventions aimed at modifying diet and encouraging greater adherence to the Mediterranean diet have shown success at improving mental health (e.g., anxiety and depression) for some, but not all, study participants [4,5]. A common hypothesis across these diet improvement studies is that nutrient density is increased through higher consumption of vegetables and fruit, and lower consumption of ultra-processed foods.

However, a number of factors may impair an individual’s ability to fully benefit from dietary manipulation alone: both environmental and individual variables. On the environmental front, the depletion of essential nutrients in our food (e.g., magnesium, selenium, copper) over the last century [6], the use of herbicides and pesticides that diminish essential nutrients in crops through chelation of minerals [7], an emphasis on high-yield crops at the expense of nutrient density [8], and even the increase in atmospheric CO_2_ [9,10] have all been identified as threats to the quality of human nutrition and contributors to the reduced nutrient density of plants.

At the individual level, several factors may contribute to the need for more nutrients than what might be available in one’s diet, even if characterized as a “healthy” diet. These factors include poor gut health and microbiome composition [11,12,13], the presence of inflammation [14], genetic variation leading to possible in-born errors of metabolism, which slow metabolic activity due to suboptimal availability of vitamin and mineral cofactors [15], and mitochondrial dysfunction that may result in decreased production of cellular energy in psychiatric disorders [13,16,17]. The presence of any of these factors may effectively reduce the availability of nutrients necessary for optimal brain health.

Bearing in mind these factors, micronutrient supplementation may be a consideration beyond diet manipulation. However, there is an ongoing debate as to whether a single or multiple ingredient intervention is appropriate [18]. The majority of studies conducted over the last 100 years in the field of psychology and psychiatry have focused on one nutrient in order to determine the active ingredient. A number of systematic reviews and meta-analyses have highlighted the concentrated effort to find the single nutrient that may alleviate symptoms associated with complex psychiatric issues such as mood [19,20,21], psychosis [22], attention-deficit/hyperactivity disorder (ADHD) [23] or antisocial behaviors [24], with most of these studies finding only modest benefit from a single nutrient approach.

Based on an improved understanding of the physiological requirements of the brain [25], researchers are acknowledging that the search for a single nutrient to resolve complex psychiatric symptoms is unrealistic. Instead, consuming a variety of essential minerals and vitamins in combination, rather than single nutrients, makes physiological sense [17].

For example, multiple nutrients are required in biological processes related to optimizing metabolic function for mental health such as the methylation and the Krebs cycles [17]. Neurotransmitter metabolism-synthesis, uptake, and breakdown-requires enzymes at each step, and the enzymes are dependent upon multiple cofactors, most of which are a variety of vitamins and minerals. These biological processes support the necessity of ingesting nutrients in combination in order to optimize brain health.

The last two decades have ushered in a slow, but progressive expansion in the number of clinical trials conducted using a broad-spectrum multinutrient approach to treat psychological symptoms, demonstrating larger effect sizes than single nutrient studies [26,27] with the effects consistently demonstrating a global benefit, while symptom-specific improvement has varied by the rater (e.g., parent, teacher, and self). These broad-spectrum formulas are based on the premise that a multinutrient approach effectively addresses the complex array of cofactor requirements for optimal brain function. To date, there have been no systematic reviews or meta-analyses considering the strength of the multinutrient approach in randomized controlled trials (RCTs) in a clinical population. As such, this systematic review and meta-analysis will evaluate the scientific literature on the use of multinutrient and broad-spectrum multinutrients for the treatment of psychiatric symptoms. *Multinutrients* are defined, for the purpose of inclusion in this paper, as formulas containing four or more ingredients: vitamins and/or minerals, with most nutrient levels at or below Recommended Dietary Allowances (RDAs), now referred to as Dietary Reference Intakes (DRIs). *Broad-spectrum* multinutrient formulas are further delineated in this paper as containing a full complement of vitamins and essential minerals, typically 20+ ingredients, many above RDA levels, but below upper tolerable limits (ULs) [28].

Historically, clinicians and consumers have turned to the RDA/DRI as a metric to gauge the sufficient amount of a nutrient needed for optimal health. Developed in 1941 by the National Academy of Medicine (formerly the Institute of Medicine (IOM) of the National Academies), the RDA/DRI is the nutrient level determined to prevent frank deficiency in an otherwise healthy population [28]. In the context of mental health, the RDA is not necessarily an appropriate metric, as RDA levels were not established to account for brain health [29]. As a highly metabolically active organ, the brain may require nutrients at levels higher than the RDA, but at doses below upper tolerable limits (ULs) or lowest observed adverse events levels (LOAELs) [28], particularly in individuals who are experiencing psychiatric symptoms.

## 2. Materials and Methods

This systematic review was prospectively registered with the National Institute for Health Research website PROSPERO. Details of the protocol can be accessed at: www.crd.york.ac.uk/PROSPERO/display_record.php?RecordID=79164.

### 2.1. Studies Included

The study selection criteria were defined before searches were completed. Only articles available in English on RCTs were included. Searching was limited to human studies. Abstracts, letters, and conference reports were searched for full-text references. There was no restriction on blinding.

### 2.2. Participants

Only studies investigating psychological or psychiatric symptoms or outcomes (e.g., acute stress, ADHD, or depression) in humans were included in the review. The selection criteria required that participants were admitted to this study with elevated symptoms on at least one psychological measure, or this study required a psychiatric diagnosis or presentation (e.g., Major Depressive Disorder) for enrollment. Trials in asymptomatic or healthy populations, or trials focused solely on improving cognition, learning or achievement in the absence of a deficit were excluded. Trials on populations with dementia, without externalizing or internalizing behavioral concerns (e.g., aggression, mood, and anxiety) were excluded, as the topic area deemed was appropriate for a separate review. No other exclusion criteria applied (e.g., population age, gender, and sample size).

### 2.3. Interventions

The selection criteria required that the formulas studied must consist of at least four vitamins and/or minerals. Formulas containing botanicals, amino acids and essential fatty acids (EFAs) were included only if part of a formula or treatment alongside at least four vitamins and/or minerals. A minimum of four was chosen: (1) to capture, for example, the B vitamins essential for the methylation cycle and important for brain health [30] (though studies did not have to contain four B vitamins); (2) to measure the impact of nutrients used as a combination intervention, in contrast to an ’add on’ to another nutrient of interest (e.g., EFAs). Formulas without complete information on specific ingredients (including dose) were excluded (e.g., an herbal formula [31] containing vitamins did not specify ingredients or dose). In this review, the term “multinutrients” is defined as vitamins and minerals, given their importance as necessary cofactors for neurotransmission. No restrictions were placed on intervention length. Studies were required to provide information on treatment responses. Formula brand names were listed when provided.

### 2.4. Comparators/Control

All control treatments were included: placebos, non-treatment controls (e.g., waitlist), and active controls (e.g., single-nutrient supplements, medications or psychological therapies).

### 2.5. Outcome Measures

Only studies that used a psychological measure of psychiatric symptoms (e.g., acute stress, mood, and anxiety) were included. Studies that used measures of cognition/cognitive decline/dementia only (without assessing other psychological symptoms such as mood) were excluded. Studies were included regardless of whether the psychological measure was a primary or secondary outcome measure. As per published consensus guidelines [32], authors were inclusive in primary analyses and then explored the sources of heterogeneity, including clinical heterogeneity in subsequent sensitivity analyses [32]. This review’s primary outcome was a change in score on measures of psychological symptoms (e.g., mood, anxiety, and inattention) from baseline to end of intervention period (or last follow up).

### 2.6. Search Strategies for Identification of Studies

#### 2.6.1. Electronic Searches

Relevant studies were identified through literature searches using MEDLINE, PsycINFO, PsycARTICLES, the Cochrane Central Register of Controlled Trials (CENTRAL) and Google Scholar, for studies published up to 31 May 2019. There was no cut-off date. The searches were conducted using Medical Subject Headings (MeSH) terms where possible and included vocabulary related to micronutrients (e.g., vitamin and mineral), as well as psychological or psychiatric disorders (e.g., autism and ADHD); search strategies were adapted as appropriate for the database. See Appendix A for specific search strategies.

#### 2.6.2. Other Sources

To minimize publication bias, grey literature was searched. Sources including doctoral dissertations, clinical trials registries: the Australian New Zealand Clinical Trials Registry, anzctr.org.au, the European Union Clinical Trials Register, clinicaltrialsregister.eu, and the National Institutes of Health, United States-based ClinicalTrials.gov were searched for any unpublished trials, and the authors were contacted for results. Reference sections from relevant articles were examined for additional resources and included if a study met the criteria.

### 2.7. Selection of Studies

The Preferred Reporting Items for Systematic Reviews and Meta-Analyses (PRISMA) process was used for study selection. Study identification was conducted by two authors independently and in duplicate (Jeanette Johnstone, Andrew Hughes); discrepancies were discussed and resolved by consensus. Both authors read full abstracts of studies if either rater judged this study to be potentially relevant. If doubt existed, the article was included for the next stage of evaluation. After the screening, all remaining full-text articles were independently screened by the larger team of authors for possible inclusion. Agreement between the raters was monitored using Covidence v2.0, Veritas Health Innovation, Melbourne, Australia, www.covidence.org, and is reported below. Any disagreement was resolved by discussion. The authors of this review were not blind to the authors, journals, results, or conclusions of the included studies.

### 2.8. Data Extraction

Data from the included studies were extracted by a pair of reviewers independently and in duplicate (Andrew Hughes, Amy Romijn, Jeanette Johnstone) to maximize accuracy. Coded information was extracted using Covidence, and included population, sample size, number of participants in each group, intervention, control product, length of intervention period, and psychological outcome measures used.

### 2.9. Assessment of Methodological Quality of Included Studies

Studies were scored independently by a pair of reviewers (Andrew Hughes, Amy Romijn, Jeanette Johnstone) using the Cochrane risk of bias tool [33] to assess risk of bias across the following domains: sequence generation, allocation concealment, blinding of participants and personnel, blinding of outcome assessment, incomplete outcome data, selective reporting, and other risk of bias. Any reviewer disagreement was resolved by consensus and recorded using Covidence.

### 2.10. Data Synthesis and Measures of Treatment Effect

We planned to be cautious when deciding when to pool across studies due to the presumed high level of heterogeneity inherent in this type of study design. Effect sizes were computed using parameters taken from study reports including means and standard deviation (SD) or standard error (SE); correlation coefficients; odds ratios (OR) or a regression coefficient with an N or 95% Confidence Interval (CI); *p*-value with an N. Effect estimates for continuous outcomes were reported using mean differences or standardized mean differences as appropriate. Because not all statistically significant differences are large enough to be clinically meaningful, findings are presented alongside established or estimated minimal clinically important differences (MIDs) to aid in clinical interpretability. Published MIDs were used if available. If unavailable, authors followed published guidance and used 0.5 SD as an estimate of MIDs [34].

### 2.11. Missing Data

Reporting of per-protocol (completer) data, rather than intention-to-treat (ITT) data, was noted in the narrative synthesis. Where studies did not present sufficient data and/or statistical test results to allow for the calculation of the effect estimate, authors were contacted. When further information was not available, the authors’ interpretation of their results was outlined in the appropriate table and narrative synthesis, noting insufficient data to calculate effect estimates.

### 2.12. Assessment of the Quality of the Effect Estimate

We used the GRADE criteria to explore the certainty in the effect estimates reported in this review. While described in detail elsewhere [35], briefly, GRADE assumes that an RCT evidence base begins as high quality, and is ranked down as appropriate by the assessment of the following five domains: (1) risk of bias of the included studies, (2) inconsistency in the effect estimate across studies, (3) indirectness of the outcome measure to the outcome of interest, (4) imprecision of the summary estimate, and (5) publication bias. GRADE rating was performed independently and in duplicate by Joshua Goldenberg and Andrew Hughes, with disagreement resolved by consensus. Based on published guidelines, we used formalized language to discuss the results based on the GRADE rating and magnitude of effect [36].

## 3. Results

### 3.1. Study Selection and Inclusion

**Figure 1:**
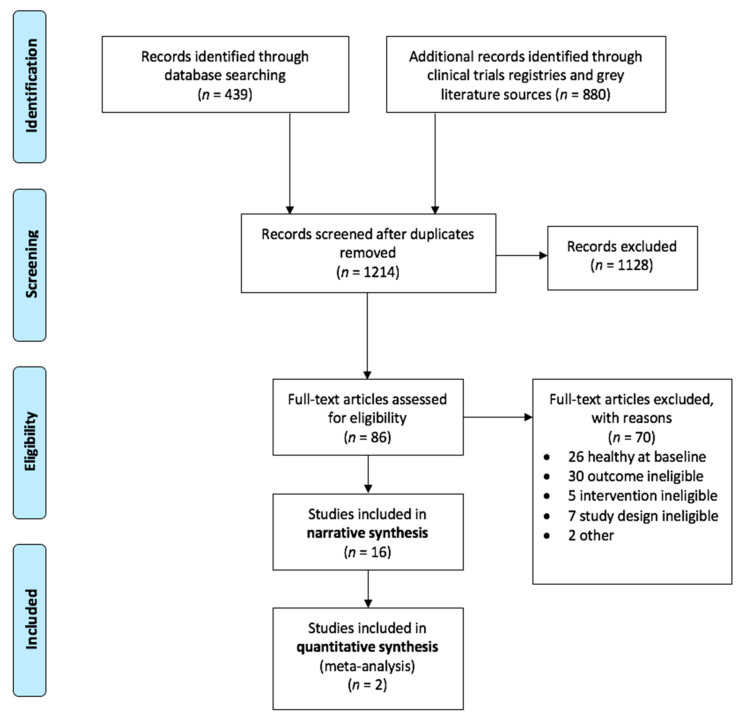
Systematic review and meta-analysis of multinutrients for psychiatric symptoms. PRISMA (The Preferred Reporting Items for Systematic Reviews and Meta-Analyses) flow diagram.

Figure 1 contains the PRISMA [37] flow diagram for study selection and inclusion. Of the 1214 abstracts screened, 16 studies met the inclusion criteria in the following six psychiatric categories: depression, post-natural disaster stress, antisocial behaviors, behavioral issues in dementia, ADHD, and autism; *n* = 1737 participants in total. Study characteristics are displayed in tabular and narrative form and described in a narrative synthesis below. If a study was registered prior to initiation, the registration is noted in the table. Because of the heterogeneity among study samples, nutrient formulas, and outcome measures, and due to unavailability of sufficient data to meta-analyze, only two studies were included in a quantitative synthesis meta-analysis [26,27].

Of the 16 studies, one study included only women [38]; one only men [39]; four included only children [27,40,41,42]; one study included adults and children [43]; and the rest only adults, both men and women [26,44,45,46,47,48,49,50,51]. Two of the studies included participants taking adjunct medications for depression [44,47]; the rest of the studies required participants to be off psychotropic medication. Two studies enrolled participants with depressive symptoms plus a genetic or inflammatory marker associated with depression [45,46]. One study added exercise and light exposure as part of the active treatment [38]. One study used vitamin D as an active control [48]. One study pooled two groups: a polyunsaturated fatty acids (PUFAs) plus multinutrients group with a PUFA-alone group and compared them to placebo. The researchers compared the PUFA groups to one another [41].

### 3.2. Formula Ingredients

**Figure 2:**
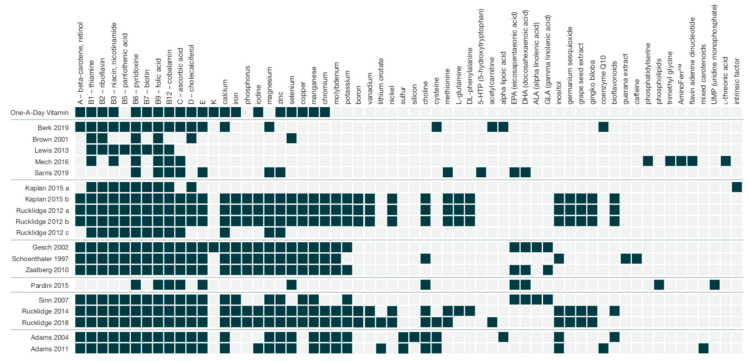
Comparing formula ingredients across studies including One-A-Day^®^, an over-the-counter formula, for comparison. AminoFerr™ contains nutritive minerals to prevent iron deficiency (specific product info. not found); intrinsic factor is a glycoprotein secreted by the stomach to aid in the absorption of vitamin B12.

**Figure 3:**
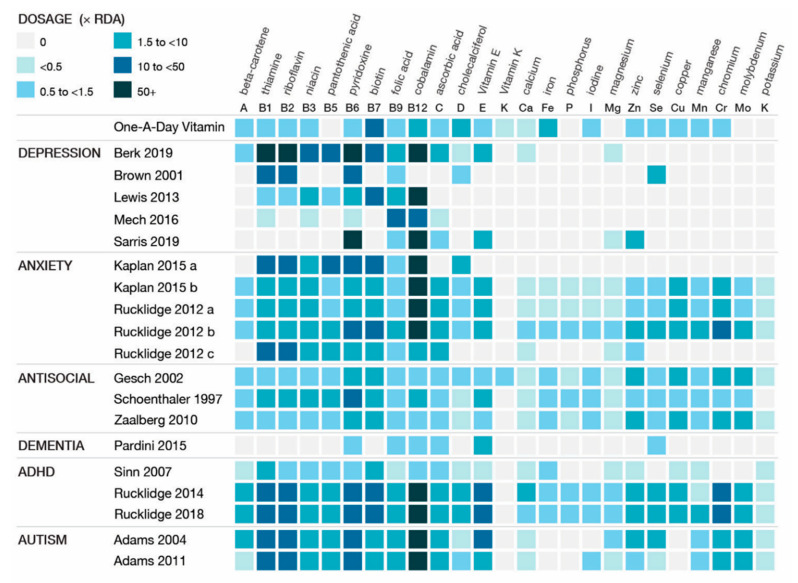
Comparing formula ingredient dosages of vitamins and minerals based on the Recommended Dietary Allowance (RDA). Kaplan, 2015 a = complex B vitamin; Kaplan, 2015 b = EMP+, 4 capsules; Rucklidge, 2012 a = EMP+, 4 capsules; Rucklidge, 2012 b = EMP+, 8 capsules; Rucklidge, 2012 c = complex B vitamins with minerals.

**Figure 4:**
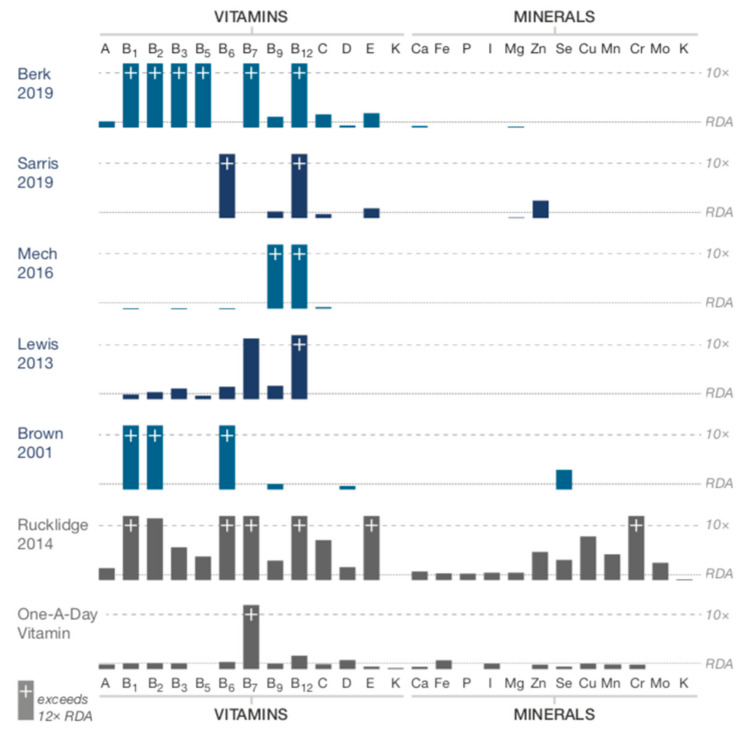
Comparing ingredient dosages in studies of participants with depression, including One-A-Day^®^, an over-the-counter formula for comparison.

**Figure 5:**
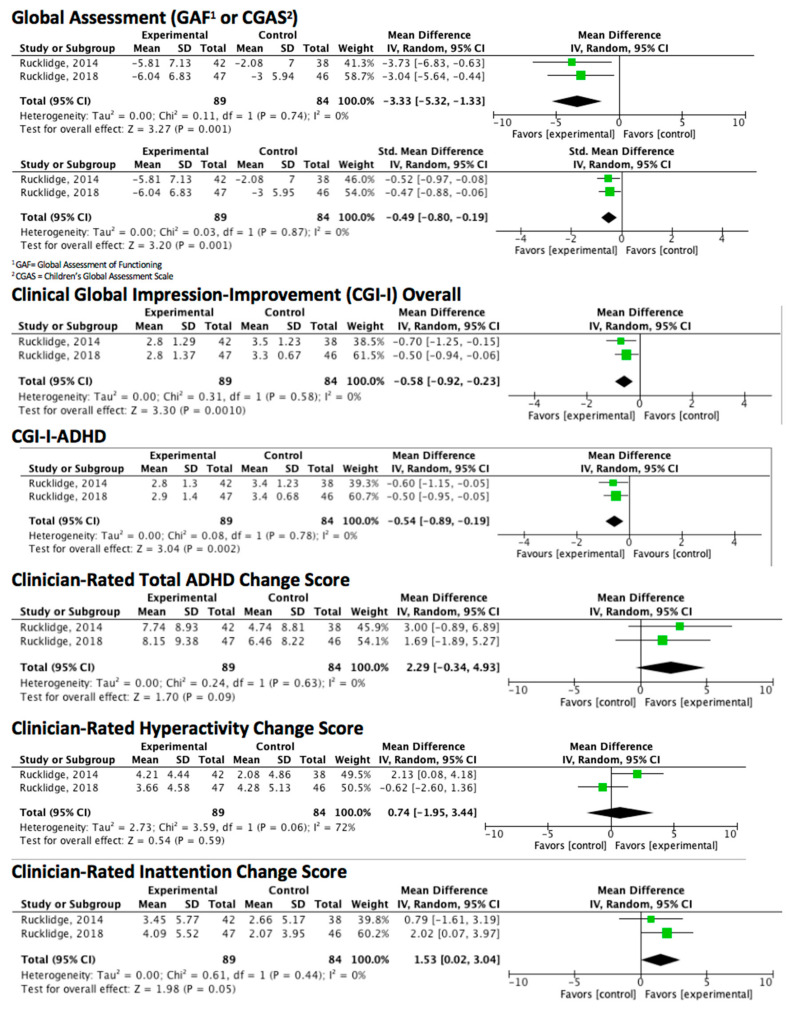

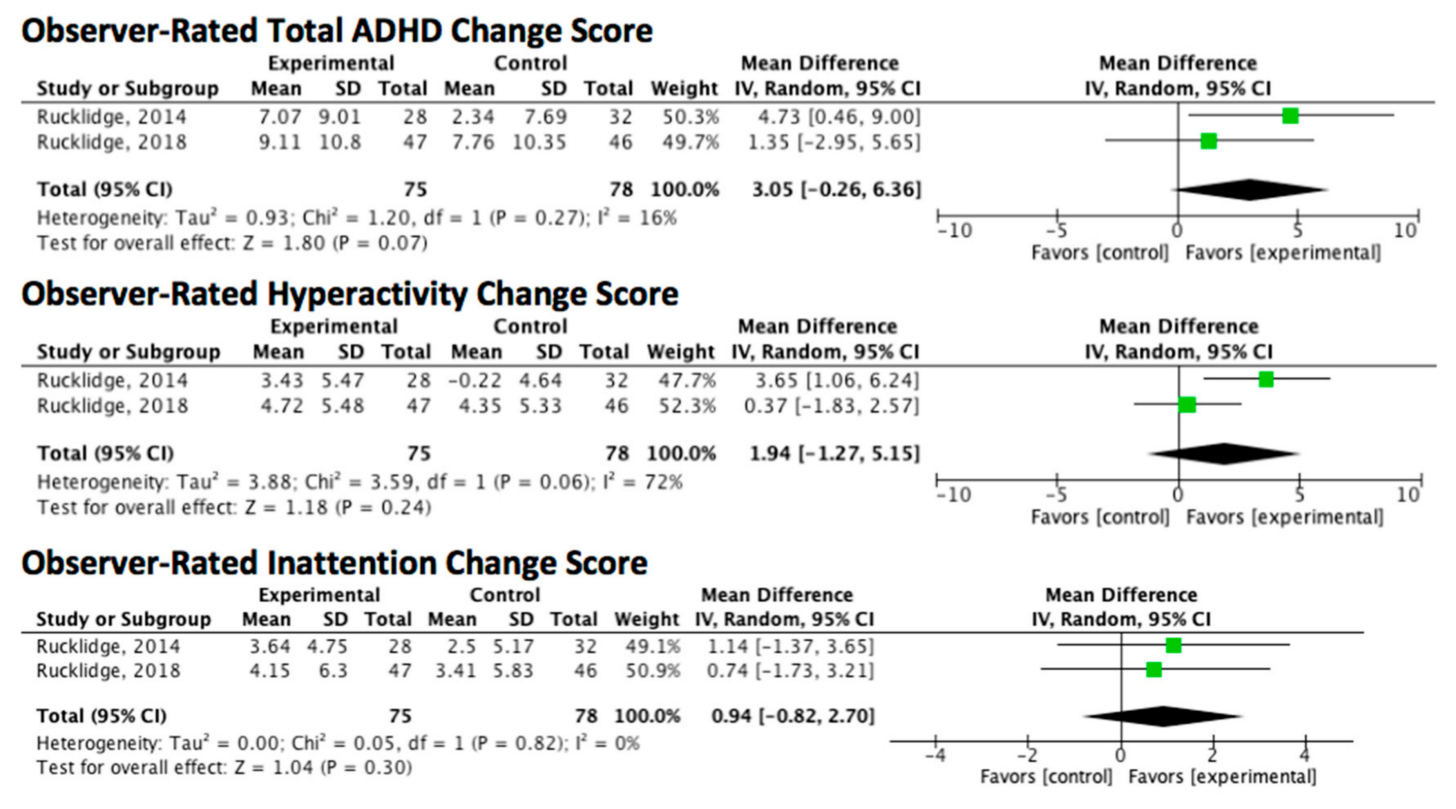
Forest plots of meta-analysis of ADHD symptoms.

**Figure 6:**
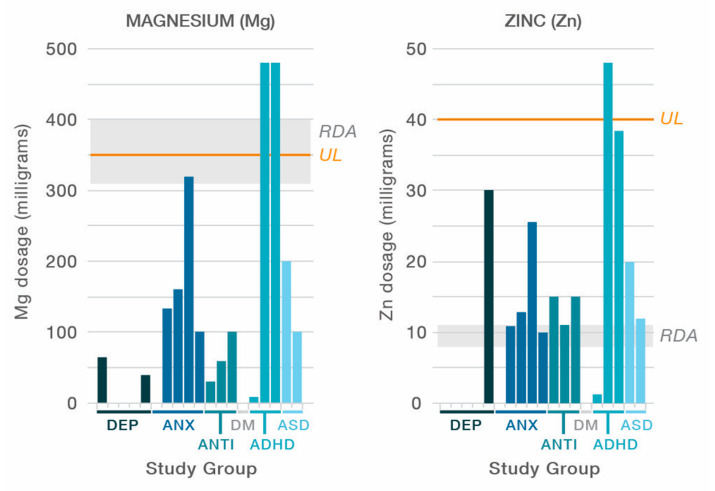
Comparing magnesium and zinc levels across studies. RDA = Recommended Dietary Allowance; UL = upper limit; DEP = depression; ANX = anxiety; ANTI = antisocial; DM = dementia behaviors; ADHD = attention-deficit/hyperactivity disorder; ASD = autism spectrum disorder.

Per the inclusion criteria, studies used formulas with ≥4 ingredients; range: 6–36. All the formulas contained vitamins B6, B9, and B12; thirteen of the formulas contained vitamin D; all the formulas except two included minerals [45,46]; range of minerals: 1–15. Ingredients and dosages for each of the formulas are listed in the tables. A visual representation of the included ingredients in each study’s formula is found in Figure 2. An over-the-counter formula, One-A-Day, is included for comparison in Figure 2, Figure 3 and Figure 4. In addition to differences between formula ingredients, the dosage of individual ingredients varied widely, with nutrients at or below the RDA, and others at levels 10–50× the RDA, but below ULs or LOAELs. For comparison across studies, the vitamin and mineral dose ranges, based on the RDA, are shown in Figure 3. Ingredients in the formulas used in the depression studies, plus a study of adults with ADHD, some of whom had moderate depression at baseline, are depicted in Figure 4. Figure 5 illustrates the forest plots of the meta-analysis. Figure 6 shows dosage comparison between formulas for magnesium and zinc, two minerals important for neurotransmission.

### 3.3. Psychiatric Categories

#### 3.3.1. Depression

Five studies investigated the impact of multinutrients on depression [38,44,45,46,47] (*n* = 707). See Table 1. The patient populations in these studies ranged from those with mild to moderate depressive symptoms [38], to adults classified with treatment-resistant Major Depressive Disorder (MDD) [47]; patients with MDD and an elevated inflammatory marker [46], or genetic polymorphism [45], and adults with bipolar disorder and current depressive symptoms [44]. Four of the studies lasted 8 weeks [38,45,46,47], and one lasted 16 weeks [44]. The key outcome for depression improvement was measured using the following instruments across the studies: the Montgomery-Asberg Depression Rating Scale (MADRS) [52], the Beck Depression Inventory (BDI) [53], and the Center for Epidemiologic Studies Depression Scale (CES-D) [54].

Three studies utilized the MADRS [44,45,47]. However, heterogeneity in populations, the inclusion criteria, and formula variations precluded meta-analysis. Berk et al., 2019 [44] enrolled 181 participants (115 included in the completer analyses) with a diagnosis of bipolar disorder who were currently experiencing depressive symptoms and were on a stable dose of medication (antidepressant, mood stabilizer, antipsychotic, or benzodiazepine). A statistically significant improvement was not observed with multinutrients compared to placebo after 16 weeks, with a mean difference (MD) −0.4, *p* = 0.91. However, 4 weeks post-discontinuation (20 weeks after baseline), a statistically significant mean difference on the MADRS of −5.2 (*p* = 0.03) was reported in the 110 completers, which is clinically significant, based on the estimated minimal clinically important difference (MID) (range: 1.6–1.9) [55]

Mech et al., 2016 [45] enrolled 330 participants (282 included in completer analyses) with major depression and an identified methylenetetrahydrofolate (MTHFR) (*C677T* or *A1298C*) polymorphism. The authors reported a mean change score difference between the multinutrient and placebo groups of −10.7 on the MADRS, which is clinically significant based on the estimated MID (range: 1.6–1.9) [55] (between group *p*-value not provided).

Sarris et al., 2019 [47] enrolled 158 participants (113 included in the completer analyses) with MDD who were taking an antidepressant for at least 4 weeks. The observed mean difference of multinutrients over placebo of −1.75 was not statistically significant (*p* = 0.33).

Two studies utilized the BDI to compare multinutrients to placebo for adults with depressive disorders [46,47]. The populations were too heterogeneous to meta-analyze: Lewis et al., 2013 [46] enrolled participants depressed at baseline with elevated homocysteine levels, while Sarris et al., 2019 [47] enrolled treatment-resistant participants taking antidepressant medication. Lewis and colleagues studied 60 adults, and although authors reported improvements in the intervention group compared to placebo on the BDI, they also stated “effect for time by randomization was not significant,” suggesting that multinutrients did not demonstrate a statistically significant difference compared to placebo (MD −0.4, *p*-value not provided). The 113 participants studied by Sarris et al. (2019) [47] did not demonstrate a statistically significant difference for multinutrients compared to placebo (MD −4.4, *p* = 0.13).

Brown et al. (2001) [38] enrolled 104 adult women with mild to moderate depression, utilizing the CES-D to compare multinutrients to placebo in intention-to-treat (ITT) analyses. A statistically, but not clinically, significant difference after 8 weeks was observed (MD −3.1, *p* = 0.004, estimated MID = 4.0) [34].

For the outcome of clinical improvement in depression, we rated the overall quality of the evidence base (GRADE) for multinutrients to be low. We rated down once due to inconsistency because the effects were not consistent across studies and populations, and once for imprecision, because we were unable to pool effect estimates across studies, and individual study estimates were imprecise. See Table 2, GRADE-Summary of Results. Overall, multinutrients **may improve** symptoms in individuals with bipolar and current depressive symptoms taking medication after 20 weeks (not significant at 16 weeks). In women with mild to moderate depression, multinutrients **may improve** symptoms **slightly**. In other studies (Lewis et al., 2013 [46], Mech et al., 2016 [45], Sarris et al., 2019 [47]) multinutrients **may have little or no difference** in outcome, though insufficient data were available to enable exact qualification [45,46].

#### 3.3.2. Post-Natural Disaster Stress

Two unblinded randomized studies examined the effect of broad-spectrum multinutrients on acute post-natural disaster stress [48,49] (*n* = 147, ITT analyses), with treatment ranging from 4 to 6 weeks. See Table 3.

Rucklidge at al. 2012 [49] included 91 adults experiencing elevated symptoms of depression, anxiety or stress after the 2011 earthquake in Christchurch, New Zealand, while Kaplan et al., 2015 [48] examined the impact of multinutrients in 56 adults experiencing elevated symptoms of depression, anxiety or stress after the 2013 floods in Alberta, Canada. Both studies used the Depression Anxiety and Stress Scale (DASS) [56] as a primary outcome measure; and for ethical reasons, both studies used active-only control treatments, no placebo. Rucklidge [49] compared two different doses of EMP+^TM^ (4 or 8 capsules, daily), a broad-spectrum multinutrient formula containing 36 ingredients, to Berocca^TM^, a B complex, which also contains minerals, as an active control [49]. Kaplan et al., 2015 compared EMP+ with a B complex and used vitamin D alone as an active control [48]. Methodological heterogeneity involving the use of all active treatments in the Rucklidge [49] study precluded meta-analysis. Both studies reported significant within-group reductions in symptoms among all groups in the studies. Rucklidge [49] found no significant differences between the B complex and EMP+ on primary outcomes, as measured by the DASS. However *post-hoc* analysis indicated that high-dose EMP+ showed significantly greater clinical improvement over the B complex control intervention, as measured by a modified Clinical Global Impression-Improvement rated by the participants on subscales for mood (*p* < 0.05) and anxiety (*p* < 0.05). Kaplan [48] reported that EMP+ and B complex were significantly more effective than the single nutrient vitamin D comparator [47]. Compared to vitamin D, the EMP+ group had greater improvement on the DASS subscales for anxiety (*d* = 1.08, 95% CI = 0.37–1.79, *p* < 0.05) and stress (*d* = 0.88, 95% CI = 0.19–1.58, *p* < 0.05) and the B complex group also had greater improvement on anxiety (*d* = 0.89, 95% CI = 0.22–1.57, *p* < 0.05) and stress (*d* = 0.76, 95% CI = 0.10–1.43, *p* < 0.05).

We rated the overall quality of the evidence base (GRADE) for multinutrients for post-natural disaster stress to be low. We rated down once for imprecision because we were unable to pool effect estimates across studies, and individual study estimates were imprecise. We rated down once for risk of bias as both studies were unblinded. See Table 2. Overall, multinutrients and B complex **may improve** symptoms of mood, stress and anxiety following a natural disaster based on self-report, with a possible additional benefit of multinutrients in improving mood and anxiety over B complex.

#### 3.3.3. Antisocial Behaviors

Three studies investigated the impact of multinutrients on offending behaviors in incarcerated populations, two in adults: Zaalberg et al., 2010 and Gesch et al., 2002 [39,50], and one in adolescents: Schoenthaler et al., 1997 [40] (*n* = 445); all used completer analyses. Trial length ranged from 2 weeks to 9 months. All three studies used a broad-spectrum multinutrient approach, with the number of ingredients ranging from 18 to 29, provided at or above RDA levels. See Table 4.

Two studies [39,50] investigated the effect of multinutrient supplementation compared to placebo on disciplinary incidents per 1000 prison days in populations of adult prisoners (*n* = 393). Due to insufficient data (lacking CIs or group percentages), studies could not be combined for meta-analysis. Gesch [50] reported that 177 participants receiving the active intervention committed 26.3% fewer offences (95% CI 8.3–44.3%; *p* = 0.03) than the placebo group, but insufficient data were provided to calculate between group differences. Zaalberg [39] (*n* = 221) also reported a reduced number of incidents in the active group (*p* = 0.017) and an increase in the placebo group. Zaalberg [39] reported non-significant effects of multinutrients compared to placebo on the Social Dysfunction and Aggression (SDAS) and the Symptom Checklist 90 (SCL-90) scales.

Schoenthaler et al., 1997 [40] investigated the effect of multinutrient supplementation on the number of violent rule infractions in a population of incarcerated individuals (*n* = 62) aged 13–17. The multinutrient group observed a decrease in mean rule violations per subject of 2.85 in the treatment arm, compared to 1.63 in the placebo arm, a difference which was statistically significant (*p* = 0.005).

We rated the overall quality of the evidence base (GRADE) of multinutrients for antisocial behaviors to be low. We rated down once for imprecision because we were unable to pool effect estimates across studies and individual study estimates were imprecise. We rated down once for risk of bias as blinding was broken in Zaalberg [39] and was assessed as having a high risk of bias. See Table 2. Overall, multinutrients **may improve** offending behaviors in incarcerated individuals.

#### 3.3.4. Behavioral Issues in Dementia

One study, Pardini et al., 2015 [51] investigated the use of multinutrients or placebo for the behavioral variant of frontotemporal dementia (bv-FTD) in 26 adults, aged 50–65 years, for twelve weeks, with a crossover to the other intervention for another 12 weeks. Participants were required to have a previous bv-FTD diagnosis for inclusion. See Table 5. Measures included the Clinical Global Impression-Severity (CGI-S) and the Neuropsychiatric Inventory (NPI). A statistically and clinically significant effect of multinutrients compared to placebo was found using the CGI-S (*p* < 0.01, MD −1.15), which was larger than the estimated MID of 0.5 [34]. However, using the NPI instrument, this study suggests a statistically significant, but not clinically significant effect (MD −4.70, *p* < 0.01, estimated MID −8.2) [57].

We rated the overall quality of the evidence base (GRADE) to be low. We rated down twice for imprecision as there was only a single study with very few participants (Table 2). Overall, multinutrients **may improve** symptoms related to bv-FTD as measured by clinician-rated severity and **may slightly improve** symptoms as measured by the NPI.

#### 3.3.5. ADHD

Three studies investigated the impact of broad-spectrum multinutrients in populations with ADHD: two in children [27,41] and one in adults [26]. See Table 6. Two Rucklidge studies [26,27] were sufficiently homogenous for meta-analysis and examined global and ADHD symptom improvement in 173 patients (80 adults, 93 children). Global assessments were measured with the Global Assessment of Functioning (GAF) scale for adults [58], and the Children’s Global Assessment Scale (CGAS) [59]. Global improvement was measured using the CGI-I overall and the Clinical Global Impression-Improvement-ADHD scale (CGI-I-ADHD). ADHD symptom improvement was measured with the Connors’ Rating Scales (CRS) and the Connors’ Adult ADHD Rating Scale (CAARS).

Based on the meta-analysis, there were statistically and clinically significant improvements on global functioning on the GAF and the CGAS (MD −3.3, *p* = 0.001, MID −3.26; SMD −0.49 *p* = 0.001 MD −0.5); and clinically and statistically significant improvements in clinician-rated CGI-I overall scores (MD −0.58, *p* = 0.001, MID −0.5), CGI-I ADHD scores (MD −0.54, *p* = 0.002, MID −0.5) and the Clinician-rated ADHD Inattention Change Score (MD −1.53, *p* = 0.05, MID −0.5). No effect was found for clinician-rated total ADHD change or hyperactivity scores. No effect was observed for pooled observer-rated ADHD scores. However, ADHD symptom improvement was clinically and statistically significant in the study of adults when measured by participant-report (MD −6.71, *p* = 0.009, estimated MID −5.4) [34]. See Figure 5.

A post-hoc sensitivity analysis revealed that 21 adults in the Rucklidge et al., 2014 study [26] met the criteria for moderate depression at baseline (MADRS score >/= 20). Participants in the micronutrient group demonstrated greater change in symptoms compared to those in the placebo group: micronutrient group: *n* = 11, MD = 9.5 standard error (SE) = 2.7; the placebo group: *n* = 10, MD = 5.1 SE = 2.0, *p* = 0.039, effect size (ES) = 0.64.

A third study, Sinn and Bryan, 2007 [41], compared the effects of two groups combined (a polyunsaturated fatty acids (PUFAs) plus multinutrients group and a PUFA-alone group) to placebo among 87 children with ADHD (completers’ analyses). Based on the Connors’ Parent and Teacher Rating Scales, the combined PUFA groups demonstrated significant improvements compared to placebo on parent ratings of inattention, hyperactivity/impulsivity, and ADHD index scores, but not teacher-rated scales. However, there were no group differences between the PUFA + multinutrients group compared with PUFA alone. Multinutrients alone were not compared to placebo. See Table 6.

We rated the overall quality of the evidence base (GRADE) for global/symptomatic improvement in ADHD to be low. We rated down once for inconsistency as there was considerable variation in the estimated effect between the different study instruments and raters. We rated down once for imprecision as the total sample size did not meet the optimal information size, See Table 2. Overall, depending on the formula used, multinutrients **may improve** ADHD symptoms based on clinician-rated global functioning and inattention, and self-report ADHD measures, but not observer-rated measures, or clinician-rated hyperactivity (Rucklidge et al., 2014 and 2018). In one study [41], insufficient data were reported to enable exact qualification of multinutrient outcomes.

#### 3.3.6. Autism

Two studies, one in children and adults with autism, Adams et al., 2011 [43] (*n* = 104), and one in children with autism, Adams and Holloway, 2004 [42] (*n* = 20), both three months in duration, examined a broad-spectrum multinutrient formula containing 29 ingredients, at doses typically higher than the RDA. See Table 7. Enrollment required a prior diagnosis of autism, pervasive developmental disorder/not otherwise specified, or Asperger’s syndrome by a psychiatrist or other clinical professional.

Clinical improvement in autism was based on the Parent Global Impressions (PGI)-Revised scales. Among the 20 children with autism, multinutrients demonstrated a statistically and clinically significant effect on the PGI sleep subscale (MD = 1.1, *p* = 0.03, MID −0.5) [42]. Among 104 children and adults with autism, multinutrients demonstrated a statistically significant (all *p*-values ≤ 0.02), but not clinically significant difference on the PGI overall, hyperactivity, tantrumming, and receptive language subscales, based on the CGI-I MID of 0.5 (MD = 0.33, *p* < 0.01) [34]. While the studies were adequately homogenous for pooling, insufficient data were available for meta-analysis. See Table 2.

We rated the overall quality of the evidence base (GRADE) for global impression in autism to be low. We rated down once due to inconsistency because the effects were not consistent across studies and populations. We rated down once for imprecision, because we were unable to pool effect estimates across studies and individual study estimates were imprecise. See Figure 2. Overall, multinutrients **may slightly improve** some autism outcomes.

## 4. Discussion

This paper systematically reviewed the RCT literature using multinutrient formulas containing vitamins and minerals as treatment for psychiatric symptoms in clinical populations. Sixteen studies were identified across six clinical areas (depression, post-disaster stress, antisocial personality behaviors, and behaviors in dementia, ADHD, and autism), with one to three studies within each category, except for depression, with five studies. Given the heterogeneity of the study formulas, populations, outcome measures used, and the absence of complete data reporting for both the active and placebo groups, only the ADHD domain could be meta-analyzed. The overall quality of evidence (GRADE) for the six areas ranged from very low to low. The limited number and small sample sizes of available RCT evidence led to down rating for imprecision across all areas. Additional concerns included risk of bias and inconsistency. Despite signals of important clinical benefit emerging across several of the studies (based on MID), alongside a substantial literature showing multinutrient benefit based on experimental methodologies beyond the traditional RCT design [60], the consistency in robust positive findings for multinutrients based on RCT evidence is lacking. Four key reasons are highlighted for these inconsistencies, which could be addressed in future studies.

### 4.1. Populations and Outcomes Studied

One striking finding is how few studies have been conducted using a multinutrient approach with *clinical* samples based on the criteria from the Diagnostic and Statistical Manual (DSM). Within this review, the DSM criteria were used in four studies of MDD, three of ADHD, two of autism and one of behavioral variants of dementia. Indeed, most studies using a multinutrient approach are conducted on non-clinical samples [61]. In these populations, given the low symptom levels at baseline, smaller effects may be observed due to a floor effect, hindering the ability to detect meaningful change. Another change consideration is the outcome of interest. Given the possible biological effects of multinutrients (e.g., optimizing neurotransmitter synthesis [62], enabling adenosine triphosphate (ATP) production [63], altering the microbiome [64], the changes may be more appropriately classified as “a metabolic tune-up” [65] and reflected in global improvement, rather than treatment of a particular symptom. For example, in the two ADHD studies where meta-analyses were possible, the most robust effects were global-the GAF/CGAS ratings: ES = 0.46/0.48; the CGI: ES = 0.57, 0.46. In contrast, effects were inconsistent for the core ADHD symptoms, depending on the rater. In narrative synthesis, Adams et al.’s autism study [43] reported a global beneficial effect with a medium effect size = 0.46 and Pardini [51] showed a significant group difference on change on the CGI-Severity score. In the prison studies, the reduction in disciplinary incidents may be interpreted as a global “calming” effect observed as reduced aggression and rule breaking behavior. Aggression also improved in the child ADHD study [27], as reported by parents and teachers. Future research would benefit from focusing on the global effect of multinutrients on observable and measurable psychiatric problems, based on the DSM diagnostic criteria.

### 4.2. Dose and Range of the Ingredients

In this paper, we used two terms to refer to the formulas: “multinutrient” for those that met the inclusion criteria of a combination of at least four vitamins and minerals, typically at or below the RDA (*n* = 7) [38,41,44,45,46,47,51]; and the term “broad-spectrum multinutrients” (*n* = 9) [26,27,39,40,42,43,48,49,50] to refer to formulas that contained all or most vitamins, plus a range of minerals, at doses typically at or higher than the RDA. Of the seven studies classified as using a “multinutrient” formula (Berk [44], Brown [38], Lewis [46], Mech [45], Sarris [47], Pardini [51], and Sinn [41]), one showed clinically and statistically significant benefit over placebo on the CGI-S [50], based on data provided in the original papers. In contrast, of the nine studies classified as “broad-spectrum multinutrients” (Kaplan, Rucklidge [26,27,48,49], Gesch [50], Schoenthaler [40], Zaalberg [39], and Adams [42,43]), eight reported between-group differences favoring active treatment [26,27,39,40,43,48,49,50] across a range of psychiatric symptoms. Figure 2 and Figure 3 illustrate the widely varying range of ingredients, and their doses, between the formulas.

Two large, well-designed studies for depression [44,47] that reported full data both showed negative results for depression symptoms, and used targeted nutrients, mainly vitamins. Among the five depression studies, only four minerals (calcium, zinc, magnesium, selenium) were included in three of the studies, with levels at or just above the RDA, see Figure 3. The authors concluded that the “shot gun approach” did not show benefit, and the nutrient combination “may not have been the best” in terms of the “dose/ratio studied” [47], a sentiment shared by authors of one of the ADHD studies regarding the multinutrients’ lack of improvement beyond what was shown with PUFA alone [41]. To illustrate the nutrient combinations in depression studies, Figure 4 compares the ingredients and dosages of the six studies, plus the Rucklidge 2014 study [26] that enrolled adults with ADHD, of which 21 met the criteria for moderate to severe depression based on a MADRS score ≥ 20 at baseline [66]. For ingredient and dose comparison with over-the-counter formulas, the One-A-Day^®^ multivitamin was shown as well. In comparison to the multinutrient formulas, the *broad-spectrum* formulas contain 15 essential minerals with doses at or above the RDA. These formulas also include amino acids and antioxidants. In considering ingredient dosages, it is prudent to remember that the RDA has not been established for mental health, and higher dosages, though below ULs, may be needed for individuals experiencing mental health symptoms [29,67].

The doses of two important minerals: magnesium and zinc are compared between formulas to highlight the dose differences, as illustrated in Figure 6. Magnesium is needed for more than 300 biochemical reactions in the body. It helps maintain normal muscle and nerve function, keeps heart rhythm regular [68], contributes to bone strength, and assists with oxidative phosphorylation in the mitochondria [69]. Magnesium is also involved in energy metabolism and protein synthesis [70] and has been found to reduce central nervous system hyperexcitability in children [71,72].

Equally important, Zinc plays a role in more than 300 enzymatic processes, with many diverse biochemical roles identified including nucleic acid metabolism, neurotransmitter production, antioxidant activity, cell signaling, and brain and immune function [73,74]. Mechanisms for improved mood through zinc supplementation are extensive, including increasing brain-derived neurotrophic factor [75], gamma amino butyric acid (GABA) [76], and other neurotransmitters, and improving the integrity of the gastrointestinal tract and epithelial junctures [77]. Zinc also acts as an antioxidant and plays anti-inflammatory roles [78,79].

### 4.3. The Use of Medications

Two of the depression studies, neither of which showed benefit on the primary outcome, included participants currently taking medications. Both studies were hampered by a large placebo effect [44,47]. Drug–drug interactions can occur when one drug impacts the absorption, transportation, metabolism, or excretion of another drug (pharmacokinetics). A drug–drug interaction may also occur when one drug affects how the body responds to another drug (pharmacodynamics), potentially without changing the second drug’s pharmacokinetics. Constituents in food and dietary supplements can also significantly change the pharmacokinetics or pharmacodynamics of drugs (e.g., St. John’s Wort and grapefruit juice inhibiting and inducing cytochrome P450 3A4 (CYP3A4), respectively) [80].

It is unknown whether a specific multinutrient dietary supplement can impact the pharmacokinetics of drugs. In theory, a multinutrient supplement could affect the absorption of a drug, thereby decreasing or increasing its bioavailability. By increasing bioavailability, there is an increase in total drug exposure that could lead to symptom development. Inversely, by reducing bioavailability, a previously therapeutic dose may become subtherapeutic due to the decrease in drug absorbed. Another possible multinutrient-drug interaction could occur at the pharmacodynamic level, particularly with psychiatric medications.

A multinutrient supplement may ensure that adequate quantities of essential cofactors are available to synthesize neurotransmitters in the brain, gut, or both. In theory, supplementation may lead to an increase in neurotransmitter synthesis when adequate quantities of these cofactors, based on a specific individual’s needs, are not obtained from the diet. An increase in neurotransmitter synthesis may change how an individual responds to a medication whose mechanism targets the same neurotransmitter. A meta-analysis of several individual micronutrient supplements, used in combination with antidepressants, showed improved response to antidepressants, supporting the potential of pharmacodynamic interactions [19]. A study of adjunctive treatment for psychosis with multinutrients demonstrated benefit at one-month, continuing up to two years, compared to patients who took antipsychotics alone [81]. Studies examining the symptom profiles of individuals taking multinutrient supplements with medications have observed benefit when reducing medication dosages after taking a therapeutic dose of the supplement [82]. While several gaps exist in the mechanistic understanding of combining multinutrient supplements with drugs, observational data support the need for careful monitoring of therapeutic responses when combining any nutraceutical with a pharmaceutical to ensure adequate patient response. Supplementation with a multinutrient formula may require a dose reduction to avoid symptoms due to pharmacodynamic interactions. The long-term safety of multinutrient use has been observationally studied (*n* = 35) for up to 12 years of use (average = 3.75 years (SD = 3.63), with a range of 0.83–12 years), as demonstrated by blood work and participant responses [83].

### 4.4. Robustness and Breadth of Methodology

Finally, the quality of the studies, as well as the quality of the results reported, bears comment. A number of the studies in this review reported results in a format that was not conducive to meta-analytic analyses. Some studies excluded key between-group comparisons such that clear determination of a significant group difference was not possible. Many studies included per-protocol data only, rather than intention to treat. Means and standard deviations, effect sizes, and confidence intervals were inconsistently reported. These data deficiencies led to unclear and imprecise reporting of outcomes and resulted in down rating the confidence in the effect estimates. Without these data, several studies that could have been meta-analyzed, were unable to be compared, leaving a narrative summary. In some cases, authors reported active intervention benefit without presenting data to support their finding.

Moving forward, in order to optimize the opportunity to enhance the operation of *all* metabolic pathways to improve mental health, study formulas are needed that use the full array of vitamins *and minerals*. To allow for possible replication of the global improvement in psychological functioning observed in several studies, including a measure of global functioning is important. A cost-effectiveness study would enable the evaluation of a multinutrient approach relative to other treatment methods. Consideration of optimal study length is also necessary in order to capture treatment effects, if they do exist. A placebo run-in prior to study initiation may reduce placebo response.

Given the increasing number of people experiencing psychological distress [84], and the substantial number not benefitting from current treatment approaches, or not utilizing talk therapies, [85] rigorously exploring alternatives, such as the potential of multinutrients, is important. Well-powered, well-designed, and well-reported trials are needed to confidently determine whether a broad-spectrum multinutrient approach is a viable alternative, or complement, to the current psychiatric treatment regimes.

## 5. Conclusions

Compared to multinutrient formulas with fewer ingredients delivered at lower doses, broad-spectrum multinutrients demonstrated more consistent benefits for a range of mental health issues. Complex B vitamins with minerals showed benefit for symptoms of acute anxiety and stress post-natural disaster. In order to accurately understand the role of multinutrients for psychiatric symptoms, studies need to be conducted in populations with mental health issues, using the DSM criteria, looking at global outcomes. Future studies may build upon these nascent meta-analyses by reporting full data (between-group effects, confidence intervals, and effect sizes), studying similar populations, using similar formulas that contain a full range of vitamins and minerals at therapeutic doses, and the same or related global outcome measures. In studies that include participants using psychiatric medication, consideration of cross-tapering may improve outcomes.

## Figures and Tables

**Table 1:** Depression Studies (*n* = 707).

Reference	Intervention-Daily Dose	Sample Size	Sample Characteristics	Study Length	Outcomes	Results
Berk et al., 2019 [44] ACTRN12612000830897	Combined Treatment (**CT**): *N*-acetylcysteine (**NAC**) 2000 mg, Acetyl L-carnitine (ALC) 1000 mg, Ubiquinone (Co Q10) 200 mg, magnesium 64 mg (as orotate 500 mg), calcium ascorbate dehydrate 242 mg (equiv ascorbic acid 200 mg), cholecalciferol 12.5μg (equiv Vit D3 250 IU), α-tocopherol 60 IU (equiv natural Vit E 50 IU), alpha lipoic acid 150 mg, Retinyl palmitate 900 μg (equiv Vit A 3000 IU), Vit H 600 μg, thiamine hydrochloride 100 mg, riboflavin 100 mg, nicotinamide 200 mg, calcium pantothenate 100 mg, pyridoxine hydrochloride 100 mg, folic acid 800 μg, and cyanocobalamin (Vit B_12_) 800 μg ***or:*** NAC 2000 mg ***Plus:*** usual medication	*n* = 148 47 CT 52 NAC 49 placebo based on analysis	Adults with bipolar disorder (Diagnostic and Statistical Manual (DSM)-IV-TR) with current depressive episode based on Montgomery-Asberg Depression Rating Scale (**MADRS**) >/= 20; intervention was adjunctive to usual medication; multisite study	16 weeks	**Primary**: Montgomery-Asberg Depression Rating Scale (**MADRS**) **Secondary**: Beck Depression Rating Scale (**BDRS**); Young Mania Rating Scale (**YMRS**), Clinical Global Impression-Improvement (**CGI-I**) and **CGI**-Severity (**CGI-S**) subscales; Social and Occupational Functioning Assessment Scale (**SOFAS**), Longitudinal Interval Follow-Up Evaluation - Range of Impaired Functioning Tool (**LIFE-RIFT**), and Quality of Life Enjoyment, and Satisfaction Questionnaire Short Form (**Q-LES-Q**)	**Negative**: no between group differences at study end (week 16) **Positive**: at 4 weeks post-continuation (week 20; *n* = 32 for CT; *n* = 37 for placebo) improvements were significantly greater in the CT group compared to placebo on the MADRS (*d* = 0.53), BDRS (*d* = 0.50), CGI-I (*d* = −0.43), SOFAS (*d* = −0.55), LIFE-RIFT (*d* = 0.53). Authors unclear on whether improvement reflects delayed benefit or upon withdrawal from intervention.
Brown et al., 2001 [38]	B_1_ 50 mg, B_6_ 50 mg, B_2_ 50 mg, B_9_ 400 µg, Se 200 µg, Vit D 400 IU ***Plus***: 20 min walk outside, 5 days/week, with 60% target heart rate increase; and increased exposure to light	*n* = 104 53 intervention 51 placebo	Adult women, with mild to moderate depressive symptoms >/= 16 based on the Center for Epidemiology Studies Depression Scale	8 weeks	**Primary**: Center for Epidemiology Studies Depression Scale (**CESD-D**) **Secondary**: Profile of Mood States (**POMS**), Depression-Happiness Scale (**DHS**), Rosenberg Self-Esteem Scale (**RSE**), General Well-Being Schedule (**GWB**)	**Positive**: intervention group improved significantly more than placebo group in mood CESD-D (*d* = −0.32*); DHS (*d* = 0.33); self-esteem, RSE: (*d* = −0.38*); and general sense of well-being, GWB (*d* = 0.23). *lower score = improvement
Lewis et al., 2013 [46]	**Max Stress B**: B_1_ 1 mg, B_2_ 1.6 mg, B_3_ 30 mg, B_5_ 3.3 mg, B_6_ 3 mg, B_9_ 1000 μg, B_12_ 263 µg, B_7_ 334 µg_,_ PABA, Biotin, Inositol	*n =* 60 30 intervention 30 placebo	Adults with major depressive disorder (MDD) or a related depressive disorder (DSM-IV-TR definition) and elevated level of homocysteine (>10 μmol/L)	8 weeks	**Primary**: Beck Depression Inventory-II (**BDI**) **Secondary**: Beck Anxiety Index (**BAI**); Quality of life from the Medical Outcomes Study Short Form 36 (**SF-36**)	**Unclear**: improvements are reported by the authors for the intervention group compared to placebo in depression on the BDI, anxiety on the BAI, and overall mental health on the SF-36; however, authors also report, “effect for time by randomization was nonsignificant,” suggesting no between-group differences
Mech et al., 2016 [45] NCT02709668	**EnLyte**^®^: B_9_ citrated folic acid 1 mg, folinic acid 2.5mg, l-methylfolate magnesium 7 mg, B_1_ 25 μg, Flavin adenine dinucleotide 25 μg, Pyridoxal 5′-phosphate 25 μg, B_12_ 50 μg, Nicotinamide adenine dinucleotide (NADH) 25 μg, Trimethyl glycine 500 μg, **AminoFerr** ^TM^: 1.5 mg, Vit C 25 mg, l-threonic acid 1 mg, **Sharp PS^®^ Gold**: phosphatidylserine-omega-3 conjugated 20 mg	*n =* 282 159 intervention 123 placebo	Adults with MDD (DSM-5 definition) and positive for either methylenetetra-hydrofolate (MTHFR) *C677T* or *A1298C* polymorphism	8 weeks	**Primary**: MADRS	**Unclear**: improvements are reported by authors for depression on the MADRS (*d* = −0.81) in intervention group compared to placebo, however, between-group comparisons and M(SD) are not included in the paper; lower homocysteine in intervention group (*d* = −0.88).
Sarris et al., 2019 [47] ACTRN12613001300763 and 12613001299796	S-adenosyl methionine (SAMe) 800 mg, folinic acid 500 μg, Vit B_12_ 200 μg, Omega-3 fatty acid concentrate (EPA-esters 1000 mg, DHA-esters 656 mg), 5-HTP 200 mg, zinc picolinate elemental 30 mg, Vit B_6_ 100 mg, Vit C 60 mg, magnesium amino acid chelate, elemental 40 mg, Vit E 40 IU *Plus*: current SSRI	*n* = 113 56 intervention 57 placebo	Adults with MDD who are inadequately responsive to current MDD medication and >/=18 on MADRS or >/=14 if not medicated; multisite study	8 weeks	**Primary**: MADRS **Secondary**: Beck Depression Inventory-II (**BDI-II**), Hamilton Anxiety Rating Scale (**HAMA**), Short Form Survey-12 (**SF-12**), Leeds Sleep Evaluation Questionnaire (**LSEQ**), CGI-I & CGI-S	**Negative**: placebo superior to nutraceutical combination in reducing MADRS scores (*d* = 0.21); response rates: 51% for the placebo and 40% for the active intervention; remission rates: 43% and 34% for placebo and active groups, respectively; no differences on other measures

ACTRN = Australian New Zealand Clinical Trials Registry Number; NCT = National Clinical Trial (US) number; Sample size *n* refers to the number who were included in analyses; IU = international unit, RDA = recommended dietary allowance; Cr = chromium; P = phosphorus; Se = selenium; Mn = manganese; Cu = copper; Zn= zinc; Mg = magnesium; I = iodine; Ca = calcium; Fe = iron; Si = silicon, silica; Cl = chloride; K = potassium; Vit A = beta-carotene, retinyl palmitate; Vit C = ascorbate, ascorbic acid; Vit D, D_3_ = cholecalciferol; Vit E = d-alpha tocopheryl succinate; B_1_ = thiamine, B_2_ = riboflavin; B_3_ = niacin, nicotinamide; B_5_ = pantothenic acid, B_6_ = pyridoxine; B_9_ = folic acid, folate; Vit H = biotin; EFA = essential fatty acids; EPA = eicosapentaenoic acid; DHA = docosahexaenoic acid; *d* = Cohen’s d; Primary = measure used in study for participant inclusion criteria.

**Table 2:** GRADE-summary of results.

Outcomes	Results	№ of Participants (Studies)	Certainty of the Evidence (GRADE)
Depression: Clinical Improvement (Depression) Assessed with: MADRS, CES-D, BDI Follow up: range 8–20 weeks	Five studies investigated the impact of multinutrients on depressive symptoms [38,44,45,46,47]. In two studies the benefit is unclear: one study [45] showed what appears to be a clinically significant effect in a population with both depression and an MTHFR genetic variant (MD = −10.7, MID = 1.6–1.9) but provided no between-group *p*-value; the second study [46] included a population with elevated homocysteine and reported benefit on the BDI, but statistical data did not suggest between-group differences. Another study [38] reported a statistically, but not clinically significant effect (MD = 3.1, *p* = 0.004, MID = 4). Two studies [44,47] did not show a clinically or statistically significant effect at the primary outcome endpoint.	707 (5 RCTs)	⨁⨁◯◯ LOW ^a, b^
Post-Natural Disaster Depression, Anxiety, Stress, Assessed with: DASS Follow up: range 4–6 weeks	Two studies investigated the effect of multinutrients on post-natural disaster (flood, earthquake) symptoms of depression, anxiety, and stress [48,49]. Both studies compared similar multinutrient formulations to active controls. Within group improvements were observed in both studies across all treatment groups. The flood study observed greater improvement over time with multinutrients compared to vitamin D on measures of anxiety (*d* = 1.08) and stress (*d* = 0.88), but not for depression. While there were no significant between group differences between two different doses of multinutrients and the B-complex with minerals in the earthquake study, with all three groups improving, more participants were rated as treatment responders with the multinutrient intervention.	147 (2 RCTs)	⨁⨁◯◯ LOW ^b, g, h^
Antisocial Behavior (Antisocial) Assessed with: Number of disciplinary incidents per 1000 person/days, reports of serious offenses, violent rule infarctions reported by prison staff, SDAS, GHQ 28 Follow up: range 2 weeks to 9 months	Three studies measured the effect of multinutrients on antisocial or offending behavior measured as disciplinary incidents in incarcerated populations [39,40,50]. Two studies that were sufficiently homogeneous to meta-analyze [39,50], reported greater improvements in the multinutrient group vs placebo, but provided insufficient data to perform between group comparisons. The third study [40] investigated the effect of multinutrient supplementation on the number of violent rule infarctions in a population of incarcerated individuals aged 13–17. Multinutrients demonstrated a decrease in mean rule violations per subject of 2.85, compared to 1.63 in the placebo arm, a difference which was statistically significant (*p* = 0.005). MID is unclear.	455 (3 RCTs)	⨁⨁◯◯ LOW ^d, f, g^
Behavioral issues in Dementia (Dementia) Assessed with: NPI, CGI-S Follow up: 12 weeks	For the outcome of behavioral issues in the context of dementia, one study measured the effect of a multinutrient vs placebo using the CGI-S and NPI [51]. Using the CGI-S, this study suggests a statistically and clinically significant effect of multinutrient supplementation in this population (MD = −1.15, *p* < 0.01, MID = −1.1). However, using the NPI instrument, the study suggests a statistically, but not clinically significant effect (MD = −4.70. *p* < 0.01, MID = −8.2).	26 (1 RCT)	⨁⨁◯◯ LOW ^e^
ADHD: Global/Symptomatic Improvement (ADHD) Assessed with: CGI-I, CGI-ADHD, CPRS, CAARS Follow up: range 8–15 weeks	Three studies investigated the impact of multinutrients on global and symptom improvement in patients with ADHD [26,27,41]. One study showed benefit for two pooled groups (multinutrients plus PUFA and PUFA-alone) compared to placebo, but did not find group differences between the multinutrients plus PUFA group compared to the PUFA alone group [41]. Two studies were sufficiently homogenous and were combined in meta-analyses [26,27]. The results showed clinically and statistically significant improvements on global functioning SMD = −0.49, *p* = 0.001, clinically and statistically significant improvements on clinician-rated global scores (MD = −0.58, *p* = 0.001, MID = −0.5) and ADHD scores (MD = −0.54, *p* = 0.002, MID = −0.5). Pooled analysis of clinician-rated symptom scores showed a statistically significant improvement for inattention (MD = 1.53, *p* = 0.05), but not for hyperactivity or total scores. No effect was observed for pooled observer-rated ADHD scores. ADHD symptom improvement was statistically and clinically significant in the adult study when outcome was measured by participant-report (MD = 6.71, *p* = 0.009, MID = 5.9).	260 (3 RCTs)	⨁⨁◯◯ LOW ^c, d^
Autism Assessed with: Parent Global Impression Follow up: 12 weeks	Two studies investigated clinical improvement in autism [42,43], both used the Parent Global Impression (PGI) scale. In children with autism, multinutrients demonstrated a statistically and clinically significant difference compared to placebo on the PGI sleep subscale (MD = 1.1, *p* = 0.03, MID = 0.5). In children and adults with autism, multinutrients demonstrated a statistically, but not clinically, significant difference in PGI ratings (MD = 0.33, *p* < 0.01, MID = 0.5). While the studies were adequately homogenous for pooling, confidence intervals were not consistently reported, which precluded meta-analysis.	124 (2 RCTs)	⨁⨁◯◯ LOW ^b, d^

^a^. Inconsistent results between varying populations studied; ^b^. Narrative synthesis was conducted, estimates are not precise; ^c^. Considerable variation in the estimated effect between the different study instruments and raters; ^d^. Total population does not meet optimal information size thresholds; ^e^. Only a single study with very few participants; ^f^. Blinding was broken in Zaalberg et al., 2010; ^g^. Confidence interval is wide and includes significant improvement, no effect, and worsening of effect; ^h^. Both studies were unblinded and had a high risk of bias; MD = Mean Difference; MID = Minimal Clinically Important Difference; SMD = standardized mean difference; *d* = Cohen’s d; RCT = randomized controlled trial; MTHFR = methylenetetrahydrofolate; MADRS = Montgomery-Asberg Depression Rating Scale; CES-D=Center for Epidemiologic Studies-Depression Scale; BDI = Beck Depression Inventory; DASS = Depression Anxiety and Stress Scale; SDAS = Social Dysfunction and Aggression Scale; GHQ 28 = General Health Questionnaire-28; NPI = Neuropsychiatric Inventory; CGI-S = Clinical Global Impression-Severity; CGI-I = Clinical Global Impression-Improvement; CGI-ADHD = Clinical Global Impression-ADHD; CPRS = Conners Parent Rating Scale; CAARS = Conners Adult ADHD Rating Scale.

**Table 3:** Post-natural disaster stress studies (*n* = 147).

Reference	Intervention Daily Dose	Sample Size	Sample Characteristics	Study Length	Outcomes	Results
Kaplan et al., 2015 [48] ANZCTR 12613001051730	**EMPowerplus (EMP+)**^TM^: 4 capsules containing Vit A 384 μg, Vit C 133.2 mg, Vit D 320 IU, Vit E 53.6 mg, B_1_ 4 mg, B_2_ 3.2 mg, B_3_ 20 mg, B_5_ 4.8 mg, B_6_ 8 mg, B_9_ 320 µg, B_12_ 293.2 µg, Biotin 240 µg, Ca 293.2 mg, Fe 3.2 mg, P 186.8 mg, I 45.2 µg, Mg 133.2 mg, Zn 10.8 mg, Se 45.2 µg, Cu 1.6 mg, Mn 2.0 mg, Cr 138.8 µg, Mo 32.0 µg, K 53.2 mg, plus a proprietary blend of Phenylalanine, L-methionine, Citrus bioflavonoids, Germanium sesquioxide, Nickel, Vanadium, Grape seed, L-glutamine, Inositol, Choline bitartrate and Ginkgo biloba ***or*** B-complex: B_1_ 50 mg, B_2_ 20 mg, B_3_ 50 mg, B_5_ 50 mg, B_6_ 20 mg, B_7_ 300 µg, Folate 400 µg, B_12_ 500 µg, Intrinsic factor 20 mg, ***or*** Vit D 1000 IU	*n =* 56 All active: 18 Micronutrient 17 Vit D 21 B-Complex	Adults with elevated symptoms of depression, anxiety or stress whose homes were damaged by a flood	6 weeks	**Primary**: Depression Anxiety and Stress Scale (**DASS**): Total **Secondary**: DASS Depression, Anxiety and Stress subscales; Modified Clinical Global Impression- Improvement (**CGI-I**): Mood, Anxiety, Stress subscales completed by the participants	**Positive**: The micronutrient and B-complex groups experienced significant declines in psychological symptoms compared with vitamin D alone. Micronutrient vs vitamin D: DASS: total (*d* = 0.94); depression (*d* = 0.64); anxiety (*d* = 1.08), stress (*d* = 0.88), as reported by authors. B-complex vs vitamin D:DASS: total (*d* = 0.81); depression (*d* = 0.58); anxiety (*d* = 0.89), stress (*d* = 0.76), as reported by authors. No significant differences between micronutrient and B-complex.
Rucklidge et al., 2012 [49] ANZCTR12611000460909	**CNE**^TM^ (equivalent to EMP+^TM^ as above) as a “low dose” (4 capsules) ***or*** a “high dose” (8 capsules) ***or*** 1 tablet of **Berocca**^TM^ containing Vit A 100 IU, Vit C 1000 mg, B_1_ 15 mg, B_2_ 15 mg, B_3_ 50 mg, B_6_ 10 mg, B_9_ 400 µg, B_12_ 10 µg, B_7_ 150 µg, B_5_ 23 mg, Ca 50 mg, Mg 50 mg, Zn 10 mg, Na 260 mg, Vit K 5 mg	*n =* 91 All active: 30 Berocca 31 CNE 4 capsules daily 30 CNE 8 capsules daily	Adults experiencing heightened anxiety or stress 2–3 months post-earthquake	4 weeks	**Primary**: DASS: Total **Secondary**: DASS subscales; Impact of Events Scale (**IES**); Perceived Stress Scale (**PSS**); Traumatic Exposure Severity Scale (**TESS**), modified **CGI-I**: Mood, Anxiety, Stress subscales completed by the participants	**Positive**: All three active treatment groups experienced significant reduction in psychological symptoms.

ANZCTR = Australia New Zealand Clinical Trial Registry; *n* refer to the number who were included in analyses; DBRCT = double-blind randomized controlled trial; IU = international unit; RDA = recommended dietary allowance; Cr = chromium; P = phosphorus; Se = selenium; Mn = manganese; Cu = copper; Zn = zinc; Mg = magnesium; I = iodine; Ca = calcium; Fe = iron; Si = silicon, silica; Cl = chloride; Mo = molybdenum; K = potassium; B = boron; V = vanadium; Na=sodium; Ni = nickel; Vit A = beta-carotene, retinyl palmitate; Vit C = ascorbate, ascorbic acid; Vit D, D_3_ = cholecalciferol; Vit E = d-alpha tocopheryl succinate; B_1_ = thiamine; B_2_ = riboflavin; B_3_ = niacin, nicotinamide; B_5_ = pantothenic acid; B_6_ = pyridoxine; B7 = biotin; B_9_ = folic acid, folate; Vit H = biotin; Vit K = Phytomenadione or phylloquinone; Primary = measure used in study for participant inclusion criteria.

**Table 4:** Antisocial behavior studies (*n* = 455).

Reference	Intervention Daily Dose	Sample Size	Sample Characteristics	Study Length	Outcomes	Results
Gesch et al., 2002 [50]	**Forceval**^TM^ Vit A 750 µg, Vit D 10 µg, B_1_ 1.2 mg, B_2_ 1.6 mg, B_3_ 18 mg, B_5_ 4 mg, B_6_ 2 mg, B_9_ 400 µg, B_12_ 3 µg, Vit C 60 mg, Vit E 10 mg, Vit K 120 µg, Vit H 100 µg, Ca 100 mg, Fe 12 mg, Cu 2 mg, Mg 30 mg, Zn 15 mg, I 140 µg, Mn 3 mg, K 4 mg, P 44 mg, Se 50 µg, Cr 200 µg, Mo 250 µg; ALA 1260 mg, GLA 160 mg, EPA 80 mg, DHA 44 mg	*n* = 172 82 active 90 placebo	adult prisoners (>18 years)	2 weeks to 9 months 142 days average	**Primary**: Number of disciplinary incidents per 1000 prison days; **Secondary**: Reports of serious offenses	**Positive**: Authors report the average reduction in disciplinary incidents was 35.1% for the active group compared to 6.7% for placebo group; data were insufficient in the paper to calculate effect sizes; authors also report reduction in serious offenses in active group, but not placebo group
Schoenthaler et al., 1997 [40]	Vit A 900 μg, B_1_ 3.6 mg, B_2_ 3.9 mg, B_3_ 48 mg; B_5_ 15 mg, B_6_ 30 mg, B_7_ 90 μg, B_9_ 400 μg, B_12_ 7.2 μg, Vit C 120 mg, Vit D 5 μg, Vit E 45 mg, Ca 122 mg, Fe 8 mg, K 700 mg, Iodine 0.150 mg, Mg 59 mg, Zn 11 mg, Se 55 μg, Cu 0.9 mg, Mn 2.3 mg, Chromium 35 μg, Mo 45 μg, Inositol 40 mg, Choline 40 mg, Guarana 87.78 mg, Caffeine 44 mg, p-amino benzoic acid 50 mg	*n* = 62 32 active 30 placebo	incarcerated youth (13–17 years)	12 weeks	**Primary**: Violent rule infractions reported by prison staff	**Positive**: 28% fewer rule infractions: both violent (*d* = 0.52), and non-violent (*d* = 0.70) in those who received the supplement than those who received placebo
Zaalberg et al., 2010 [39]	Vit A 875 µg, B_1_ 1.2 mg, B_2_ 1.6 mg, B_3_ 18 mg, B_5_ 4 mg, B_6_ 2 mg, B_9_ 400 µg, B_12_ 3 µg, Vit H 100 µg, Vit C 60 mg, Vit D_3_ 5 µg, Vit E 10 mg, Ca 100 mg, Mg 100 mg, P 52 mg, Zn 15 mg, Fe 12 mg, Mn 3 mg, Cu 2 mg, K 4 mg, I 140 µg, Se 50 µg, Cr 200 µg, Mo 250 µg; DHA 400 mg, EPA 400 mg, GLA 100 mg, and 2 capsules primrose oil	*n* = 221 115 active 106 placebo	adult male prisoners (18–25 years) across 8 Dutch prisons	1–3 months	**Primary**: Number of disciplinary incidents per 1000 prison days; **Secondary**: Social Dysfunction and Aggression Scale, General Health Questionnaire-28	**Positive**: Authors report 34% fewer aggressive and rule-breaking incidents vs 14% increase in the placebo group. Data were insufficient to calculate effect sizes. **Negative**: No group differences on self-reports of aggression or psychological well-being

Sample size *n* refers to the number who were included in analyses; Cr = chromium; P = phosphorus; Se = selenium; Mn = manganese; Cu = copper; Zn= zinc; Mg = magnesium; I = iodine; Ca = calcium; Fe = iron; Si = silicon, silica; Cl = chloride; Mo = molybdenum; K = potassium; B = boron; V = vanadium; Ni = nickel; Vit A = beta-carotene, retinyl palmitate; Vit C = ascorbate, ascorbic acid; Vit D, D_3_ = cholecalciferol; vit E = d-alpha tocopheryl succinate; B_1_ = thiamine, B_2_ = riboflavin; B_3_ = niacin, nicotinamide; B_5_ = pantothenic acid, B_6_ = pyridoxine; B_9_ = folic acid, folate; vit H = biotin; Vit K = Phytomenadione or phylloquinone; EFA = essential fatty acids; ALA = alpha linolenic acid, GLA = gamma linolenic acid; EPA = eicosapentaenoic acid; DHA = docosahexaenoic acid; Primary = measure used in study for participant inclusion criteria.

**Table 5:** Behavioral issues in dementia study (*n* = 26).

Reference	Intervention Daily Dose	Sample Size	Sample Characteristics	Study Length	Outcomes	Results
Pardini et al., 2015 [51]	**Souvenaid**^TM^: EPA 300 mg, DHA 1200 mg, Phospholipids 106 mg, Choline 400 mg, uridine-mono-phosphate 625 mg, Vit E 40 mg, Vit C 80 mg, Se 60 µg, B_12_ 3 µg_,_ B_6_ 1 mg_,_ B_9_ 400 µg	*n* = 26 13 active 13 placebo, crossover design	adults (50–65 years) with diagnosis of behavioral variant of frontotemporal dementia	12 weeks	**Primary**: Neuropsychiatric Inventory (NPI); **Secondary**: Frontal Assessment Battery (FAB), Clinical Global Impression-Severity (CGI-S), the Reading the Mind in the Eyes Test (RMET)	**Positive**: authors report reduced agitation, apathy, disinhibition, and irritability on the NPI; improvement on the CGI-S; an increase in Theory of Mind skills for those on active treatment; reversal of improvement when taken off active; insufficient data provided to calculate effect sizes **Negative**: no impact on executive functioning on the FAB

Se = selenium; Vit C = ascorbate, ascorbic acid; vit E = d-alpha tocopheryl succinate; B_6_ = pyridoxine; B_9_ = folic acid, folate; EFA = essential fatty acids; EPA = eicosapentaenoic acid; DHA = docosahexaenoic acid; Primary = measure used in study for participant inclusion criteria.

**Table 6:** ADHD studies (*n* = 260).

Reference	Intervention Daily Dose	Sample Size	Sample Characteristics	Study Length	Outcomes	Results
Rucklidge et al., 2018 [27] ANZCTRN12613000896774	**Daily Essential** Nutrients: Vit A 384 IU, Vit C 40 mg, Vit D 200 IU, Vit E 24 IU, Vit K 8 µg, B_1_ 4 mg, B_2_ 1.2 mg, B_3_ 6 mg, B6 4.67 mg, B9 50 μg, B_12_ 60 μg, B_7_ 72 μg, B_5_ 2 mg, Ca 88 mg, Fe 0.92 mg, P 56 mg, I 13.6 μg, Mg 40 mg, Zn 3.2 mg, Se 13.6 μg, Cu 0.48 mg, Mn 0.64 mg, Cr 41.6 μg, Mo 9.6 μg, P 16 mg. Proprietary blend: Choline bitartrate, Alpha-lipoic acid, Inositol, Acetylcarnitine (as acetyl-L-carnitine hydrochloride), Grape seed extract, Ginkgo biloba leaf extract, Methionine (as L-methionine hydrochloride), Cysteine (as N-acetyl-L-cysteine), Germanium sesquioxide (as chelate), Boron, Vanadium, Lithium orotate, Nickel. Other ingredients: Cellulose glycine 45 mg, Citric acid 26.814 mg, Magnesium stearate 24 mg, Silicon dioxide 20 mg	*n =* 93 47 active 46 placebo	Children (7–12 years) with ADHD	10 weeks	**Primary**: Conners Parent/Teacher Rating Scale-Revised (**CPRS**) Diagnostic and Statistical Manual (DSM)-IV Attention Deficit Hyperactivity Disorder (ADHD) Symptoms Total **Secondary**: Clinical Global Impression-Improvement (**CGI-I**); CGI-I-ADHD; CGI-I-Mood; Children’s-Global Assessment Scale-(**C-GAS**); Clinician ADHD-Rating Scale (RS)-IV Symptoms Total; Child Mania Rating Scale-Parent (CMRS-P); Clinician ADHD-RS-IV; Parent Strengths and Difficulties Questionnaire (**SDQ**)-Total Problem Score; Parent SDQ-Conduct Problem Score; Teacher SDQ-Total Problem score; Teacher SDQ-Conduct Problem Score Teacher Behavior Rating Inventory of Executive Function (**BRIEF**), Behavioural Regulation; Index Teacher BRIEF-Emotional Control subscale	**Positive**: CGI-I overall (*d* = 0.46) CGI-I-ADHD (*d* = 0.53); CGI-I-Mood (*d* = 0.51); C-GAS (*d* = 0.48); Parent SDQ-Conduct Problem Score (*d* = 0.52); Teacher BRIEF-Emotional Control Subscale (*d* = 0.66) **Negative**: Clinician ADHD-RS-IV Symptoms Total, CPRS DSM-IV ADHD Symptoms Total; CMRS-P; Clinician ADHD-RS-IV Conners Teacher Rating Scale-DSM-IV Total; Parent SDQ-Total Problem Score; Teacher SDQ-Total Problem Score; Teacher SDQ-Conduct Problem Score Teacher BRIEF-Behavioural Regulation Index
Rucklidge et al., 2014 [26] ANZCTR12609000308291	**EMP+**: Vit A 5760 IU, Vit C 600 mg, Vit D 1440 IU, Vit E 360 IU, B_1_ 18 mg, B_2_ 13.5 mg, B_3_ 90 mg, B_5_ 21.6 mg, B_6_ 36 mg, B_9_ 1440 µg, B_12_ 900 µg, Biotin 1080 µg, Pantothenic acid 21.6 mg, Ca 1320 mg, Fe 13.74 mg, P 840mg, I 204 µg, Mg 600 mg, Zn 48 mg, Se 204 µg, Cu 7.2 mg, Mn 9.6 mg, Cr 624 µg, Mo 144 µg, K 240 mg, Germanium sesquioxide 20.7 mg, B 2400 µg, V 1194 µg, Ni 29.4 µg, Choline bitartrate 540 mg, DL-phenylalanine 360 mg, Citrus bioflavonoids 240 mg, Inositol 180 mg, Glutamine 180 mg, L-methionine 60 mg, Gingko biloba 36 mg, grape seed extract 45 mg	*n* = 80 42 active 38 placebo	Adults with ADHD	8 weeks	**Primary**: Conners Adult ADHD Rating Scale (**CAARS**), self or observer version **Secondary**: CGI-I-Overall Impression; CGI-I-ADHD; Global Assessment of Functioning (**GAF**); MADRS; Self-report: CAARS, DSM-IV ADHD symptoms total; CAARS inattention, hyperactivity/impulsivity; Observer: CAARS DSM-IV ADHD symptoms total; CAARS, inattention, hyp/imp; Clinician: CAARS DSM-IV ADHD symptoms total; inattention, hyperactivity/impulsivity	**Positive**: CAARS DSM-IV ADHD symptoms, self-report (*d* = 0.61); CAARS ADHD symptoms, Observer (*d* = 0.59); CGI-I-ADHD (*d* = 0.53); CGI-I-Overall (*d* = 0.57); CAARS, self-report inattention (*d* = 0.62), hyperactivity/impulsivity (*d* = 0.47); CAARS, observer, hyperactivity/impulsivity (*d* = 0.67); GAF (*d* = 0.46) **Negative**: CAARS DSM-IV ADHD symptoms, clinician; MADRS; CAARS, observer, inattention; CAARS, clinician inattention, hyperactivity/impulsivity
Sinn & Bryan, 2007 [41]	**Blackmores Chewable Multivitamins & Minerals for Kids (Nutrients)**: Vit 175 IU, B_1_ 700 µg, B_2_ 1.1 mg, B_3_ 12 mg, B_5_ 2.7 mg, B_6_ 1.3 mg, B_9_ 100 µg, B_12_ 1.5 µg, Vit C 60 mg, D_3_ 100 IU, Vit E 6 IU, Vit H 50 µg, Ca 33.9 mg, Fe 7.5 mg, Mg 8.32 mg, Mn 77 µg, Cu 178.6 µg, K 118 µg *Plus*: **Polyunsaturated fatty acid (PUFA): eye q™**: EPA 93 mg, DHA 29 mg, GLA 10 mg, Vit E 1.8 mg ***or*** **eye q™** alone	*n* = 87 36 active: (nutrients + PUFA) 29 PUFA 22 placebo	Children with ADHD	15 weeks	**Primary**: Conners Parent and Teacher Rating Scales (**CPRS**), ADHD Index **Secondary**: CPRS subscales: cognitive problems/inattention, hyperactivity; Global scales for restless/impulsive, emotional liability, total; DSM-IV inattentive; hyperactive/impulsive, total; Oppositional; Anxious/Shy; Perfectionism; Social Problems; Psychosomatic	**Negative**: At 15 weeks, the PUFA group combined with the PUFA + nutrients group showed significant improvements over placebo for parent ratings of inattention, hyperactivity, and global ADHD indices on the CPRS. PUFA + nutrients compared to PUFA alone showed no group differences; authors concluded PUFA was the primary mechanism of improvement. However, the PUFA + nutrients group was not compared to placebo. No changes reported on the teachers rating scales.

ANZCTR = Australian New Zealand Clinical Trials Registry number; *n* refers to the number who were included in the analyses; IU = international unit, RDA = recommended dietary allowance; Cr = chromium; P = phosphorus; Se = selenium; Mn = manganese; Cu = copper; Zn= zinc; Mg = magnesium; Li = lithium; I = iodine; Ca = calcium; Fe = iron; Si = silicon, silica; Cl = chloride; Mo = molybdenum; K = potassium; B = boron; V = vanadium; Ni = nickel; Vit A = beta-carotene, retinyl palmitate; Vit C = ascorbate, ascorbic acid; Vit D, D_3_ = cholecalciferol; Vit E = d-alpha tocopheryl succinate; B_1_ = thiamine, B_2_ = riboflavin; B_3_ = niacin, nicotinamide; B_5_ = pantothenic acid, B_6_ = pyridoxine; B_7_ = biotin, B_9_ = folic acid, folate; Vit H = biotin; EFA = essential fatty acids; ALA = alpha linolenic acid, GLA = gamma linolenic acid; EPA = eicosapentaenoic acid; DHA = docosahexaenoic acid; Primary = measure used in study for participant inclusion criteria, Primary = measure used in study for participant inclusion criteria.

**Table 7:** Autism studies (*n* = 124).

Reference	Intervention Daily Dose	Sample Size	Sample Characteristics	Study Length	Outcomes	Results
Adams et al., 2011 [41] NCT01225198	Vit A 1000 IU, Vit C 600 mg, Vit D_3_ 300 IU, Vit E 150 IU, B_1_ 20 mg, B_2_ 20 mg, B_3_ 25 mg, B_5_ 15 mg, B_6_ 40 mg, B_9_ 100 µg, B_12_ 500 µg, Folinic acid 550 µg, Vit H 150 µg, Choline 250 mg, Inositol 100 mg, Mixed carotenoids 3.6 mg, Coenzyme Q10 50 mg, n-acetyl cysteine 50 mg, Ca 100 mg, Cr 70 µg, I 100 µg, Li 500 µg, Mg 100 mg, Mn 3 mg, Mo 150 µg, K 50 mg, Se 22 µg, S 500 mg, Zn 12 mg	*n* = 104 53 active 51 placebo	children and adults (3–58 years) with autism spectrum disorder	12 weeks	Parent Global Impressions (**PGI**)-Revised and subscales: Expressive Language Receptive language Play Cognition Sleep Sociability Eye Contact Hyperactivity Tantrumming	**Positive**: PGI-Revised subscales: Overall (*d* = 0.46) Tantrumming (*d* = 0.51); Receptive language (*d* = 0.40) Hyperactivity (*d* = 0.37) **Negative**: Expressive language, play, cognition, sleep, sociability, eye contact
Adams and Holloway, 2004 [42]	Vit A (7560, 10,584 IU), B_1_ (20, 30 mg), B_2_ (25, 25 mg), B_3_ (25, 35 mg), B_5_ (45, 25 mg), B_6_ (30, 30 mg), B_9_ (800, 800 µg), B_12_ (1200, 1600 µg), B_7_ (100, 150 µg), Choline (50, 60 mg), Inositol (50, 60 mg), Vit C (650, 800 mg), Mixed bioflavonoids (200, 400 mg), Vit D_3_ (150, 150 IU), Vit E (175, 250 IU), Ca (175, 200 mg), Ca D-glucarate (0, 75 mg), Cr (75, 100 µg), Mg (175, 200 mg), Mn (3, 3 mg), Mo (0, 75 µg), K (75, 75 mg), Se (70, 85 µg), Si (0, 3 mg), S (175, 300 mg), Zn (15, 20 mg), N-acetyl cysteine (25, 50 mg), Alpha lipoic acid (0, 25 mg)	*n* = 20 11 active 9 placebo	children (3–8 years) with autism spectrum disorder	12 weeks	(**PGI**) subscales: Sleep Gastrointestinal symptoms Expressive Language Receptive language Play Cognition Sleep Sociability Eye Contact Hyperactivity	**Positive**: PGI-Revised subscales: sleep and gastrointestinal problems; insufficient data to calculate effect sizes **Negative**: Expressive and receptive language, play, cognition, sociability, eye contact, hyperactivity

NCT = National Clinical Trials registry; *n* refers to the number who completed the study; IU = international unit; Cr = chromium; P = phosphorus; Se = selenium; Mn = manganese; Cu = copper; Zn= zinc; Mg = magnesium; Li = lithium; I = iodine; Ca = calcium; Fe = iron; Si = silicon, silica; Cl = chloride; Mo = molybdenum; K = potassium; B = boron; V = vanadium; Ni = nickel; Vit A = beta-carotene, retinyl palmitate; Vit C = ascorbate, ascorbic acid; Vit D, D_3_ = cholecalciferol; Vit E = d-alpha tocopheryl succinate; B_1_ = thiamine, B_2_ = riboflavin; B_3_ = niacin, nicotinamide; B_5_ = pantothenic acid, B_6_ = pyridoxine; B_7_ = biotin, B_9_ = folic acid, folate; Vit H = biotin; EFA = essential fatty acids; ALA = alpha linolenic acid, GLA = gamma linolenic acid; EPA = eicosapentaenoic acid; DHA = docosahexaenoic acid.

## Appendix A. Details Regarding Search Terms Used

The MeSH terms searched were “micronutri-” or “Micro-nutri-” or “vitamin” or “mineral” and “Mental disorders” or “Mental processes”. For comprehensiveness, the search was expanded to include the MeSH terms “Nutritional Physiological Phenomena” and “Mental processes” or “mental disorders”. To include studies of micronutrient deficiencies, the MeSH term “Deficiency diseases” was also included. Where MeSH terms were not able to be used, combinations of relevant search terms were used, with truncation (indicated with an asterisk) used to cover several variations of search terms (e.g., micronutrient * covers micronutrient and micronutrients). The search terms for interventions included the most commonly used terms that describe micronutrient interventions. Terms covering the most common psychiatric illnesses were used and, in line with the broad nature of the inclusion criteria of this review, the search was broadened to cover affective states and psychological outcomes. Search terms were “vitamin*” or “mineral*” or “micronutrient*” or “nutrient*” or “nutrient supplement” and “randomi*” or “trial” or “controlled study” and “mood” or “depression” or “bipolar*” or “stress” or “anxiety” or “antisocial” or “ADHD” or “autism” or “PDD” or “Asperger *” or “schizophrenia” or “psychosis” or “alcohol” or “substance use” or “smoking” or “cannabis” or “bulimia” or “eating disorder” or “anorexia”. Searches were limited to human studies and papers written or available in English. For example, CENTRAL was searched for (nutr * OR vitamin OR mineral OR micronutrient) AND (randomi * OR RCT OR placebo * OR double-blind) AND (psych * OR depressi * OR anxiety OR autism OR bipolar OR ADHD OR schizophrenia OR eating disorders OR anorexia OR bulimia OR mood OR stress OR antisocial OR aggression OR PDD OR Asperger * OR psychosis OR alcohol OR dependence OR addiction OR cannabis), with results limited to human trials.
